# Prevalence of growth monitoring practice and its associated factors at public health facilities of North Gondar zone, northwest Ethiopia: an institution-based mixed study

**DOI:** 10.1186/s12887-019-1489-4

**Published:** 2019-05-08

**Authors:** Aschilo Wubet Melkamu, Bikes Destaw Bitew, Esmael Ali Muhammad, Melkamu Tamir Hunegnaw

**Affiliations:** 1 Gondar University Hospital, Gondar, Ethiopia; 2 Department of Environmental and Occupational, Institute of Public Health, University of Gondar, Gondar, Ethiopia; 3 Department of Human Nutrition, Institute of Public Health, University of Gondar, Gondar, Ethiopia

**Keywords:** Practice of growth monitoring, Health workers, Child nutrition, Ethiopia

## Abstract

**Background:**

Growth monitoring is used to assess the growth rate of a child by periodic and frequent anthropometric measurements in comparison to a standard. However, since the practice has been poor in Ethiopia, this study aimed to assess it and its associated factors among health workers in North Gondar zone, northwest Ethiopia.

**Methods:**

An institution-based mixed study was conducted from April 1 to May 7, 2017, among 500 health workers. The multistage sampling technique was used to select participants. A structured questionnaire was used to collect quantitative data, while non-participant observation and in-depth interviews were used to generate qualitative information. Qualitative data were coded, grouped, and discussed using the identified themes. A binary logistic regression was fitted, odds ratio with a 95% confidence interval was estimated to identify the predictors of growth monitoring practice, and qualitative data were analyzed using thematic analysis.

**Results:**

Growth monitoring practice among health workers was 50.4% (95% CI: 45, 55). Work experience (AOR = 4.27, 95%CI: 1.70, 10.72), availability of growth monitoring materials (AOR = 1.52, 95%CI: 1.05, 2.20), attitude (AOR = 0.68, 95%CI: 0.47, 0.98), midwifery occupation (AOR = 0.42, 95%CI: 0.19, 0.94), and diploma level qualification (AOR = 2.20, 95%CI: 1.09, 4.45) were statistically significantly associated with growth monitoring practice.

**Conclusion:**

In this study, growth monitoring practice among health workers was lower than those of most studies. Jobs, educational status, work experience, attitude, and availability of materials were significantly associated with growth monitoring practices. Therefore, giving training to health extension and less experienced staff about growth monitoring, and providing growth monitoring equipment are important to improve health workers growth monitoring practices.

## Background

Growth monitoring (GM) is a process of regular weighing and comparing results with a standard to detect a change in growth rate irrespective of the starting height [1]. The most important issue in GM is not the position of the child on the growth curve but the direction of their growth to diagnose their health and nutritional status [2]. GM aims to improve nutritional status, reduce the risk of death or inadequate nutrition, help to educate caregivers, and lead to early referral for conditions manifested by growth disorders [1, 3].

GM has gained popularity in the last two to three decades and has been practiced in over 80 countries [4]. Currently about 154 countries, including Ethiopia, use GM as an essential element of primary health care [5]. In Ethiopia, weight charts provide a graphic representation of child weight-for-age.

Globally, 155 million under-five years of age children were stunted, 52 million wasted, and 52 million overweight [6]; in the African region, about 39.4% were stunted, 24.9% underweight and 10.3% wasted [7]. In Ethiopia, about 38% under five children were stunted, 24% underweight, and 10% wasted [8]. Ethiopia could save Ethiopian Birr 148 billion by 2025 if underweight rates are reduced to 5% and stunting to 10% in children under five years [9]. To prevent this, the National Nutritional Program of Ethiopia is considering GM as one of the strategies for improving the nutritional status of the children [10].

Although the United Nations International Children’s Emergency Fund recommends a 100% GM coverage [11], there were variations during practices in different countries. A study done in the UK showed that 64% of the respondents made at least one major mistake during the adjustment and plotting of birth weight [12]. In South Africa, health workers did not implement GM practically [13]. Even though a study in Ethiopia showed that GM was practiced in only 51% of the health facilities [14], still there were gaps in practical skills [15].

Studies indicated that various factors influenced GM practice among health workers. Studies conducted in India [16], Mangasaryan [5], and Nigeria [17] showed that training, motivation, and attitude were independent predictors of GM practice, respectively. A study done in Zambia and by the World Health Organization showed that lack of GM equipment and workload were the predictors of GM practice [18, 19], respectively. In Ethiopia, supportive supervision, logistic supplies [14], and equipment maintenance [13] were factors influencing GM practice. The above studies showed that the practice of GM was poor in different countries, including Ethiopia, and its influencing factors varied from place to place. Therefore, this study aimed to assess GM practices and associated factors among health workers in North Gondar zone, northwest Ethiopia.

## Methods

### Study design and setting

An institution-based mixed study was conducted from April 01 to May 07, 2017, in North Gondar zone, northwest Ethiopia, located 732 km from Addis Ababa, the capital of Ethiopia, to the northwest. The zone has 22 woredas (administrative divisions), ten governments and one primary private hospital, 126 public health centers, and 571 health posts. There are 2916 health professionals and 3035 health extension workers in the zone.

### Study population

The source population was all health workers practicing GM in health facilities in North Gondar zone, whereas the study population was all health workers who were responsible for GM in the selected woreda health facilities. For the qualitative component of the study, ten health facility managers were involved.

### Inclusion and exclusion criteria

All health workers in under-five outpatient departments (OPDs) in the selected woreda health facilities were included. Health workers who didn’t work in under-five OPDs were excluded.

### Sample size determination

A single population proportion formula was used to determine the sample size based on the following assumptions. The proportion of GM practice was 68.3% [20], with a 95%CI, 5% margin of error (d), 1.5 design effect, and 10% non-response rate to obtain the final sample size of 550. For the qualitative part of the study, ten health facility managers were selected purposively for an in-depth interview.

### Sampling procedure

In the quantitative part, there were 22 woredas in the zonal administration of which five were selected by using the simple random sampling technique. All of the health centers of the five wordas and all health workers who were directly involved in GM were included.

For the qualitative part, a well-structured questionnaire and an observation checklist were used as guidelines for the five health center managers’ interview and the observation of ten health facilities. The purpose of the in-depth interview and observation was to obtain general qualitative information on the importance and practice of GM and problems encountered during the process and ways of improving GM services.

### Data collection

Quantitative data were collected using a self-administered structured and validated questionnaire [13, 14] which contained socioeconomic, knowledge, attitude, and practice related characteristics. Four BSc degree graduate nurses participated in data collection. First, 22 woredas were selected from the zonal administration and then, using the simple random sampling technique, five were selected. Finally, all health centers in the five woredas were included.

For the qualitative data, the in-depth interview was used to obtain the views of the managers regarding the GM Program at their health facilities. One health manager was purposively selected from each of the health facilities.

### Data quality control

A two-day training was given to data collectors and supervisors on how to approach participants. The completeness, accuracy, and consistency of the collected data were checked every day. A pretest was administered to 55 health workers from non-selected woredas. For the qualitative component, observation was made in the morning because most beneficiaries visited facilities then. The in-depth interview was transcribed immediately after the data collection.

### Operational definitions

The dependent variable of this study was growth monitoring practice, defined as a practice of GM and following the growth rate of a child in comparison to a standard by regular, frequent anthropometric measurements in order to assess growth adequacy and identify faltering early. It was considered good practice if a health worker answered at least 75% of the 10 practice assessment questions correctly [13].

Workload: A health worker who saw 40 patient cards or more per day was regarded as very busy, 26 to 39 patient cards as busy and 25 patients or fewer per day as ideal [13].

Good knowledge, if a health worker answered at least 75% of the 12 knowledge assessment questions correctly, and favorable attitude if a health worker answered at least 75% of the 10 attitude assessment questions correctly [13].

### Data analysis

Data were cleaned and entered into EPI-Info version 7 and exported to SPSS version 20 software for further analysis. Descriptive statics and cross tabulation were carried out, and the result was presented using texts, tables, and graphs. Logistic regression was fitted to identify factors associated with the outcome variables. A predictor variable which had a *p*-value less than 0.2 in the bivariate analysis was entered into the multivariate analysis. Finally, 95%CI with *p* < 0.05 was used to declare variables which had significantly associated with the outcome variable.

Qualitative information collected through in-depth interviews and observation checklists was transcribed and translated to English before it was analyzed manually and thematically. The data-analysis process was followed by a sequence of interrelated steps, such as reading, coding, displaying, reducing and interpreting. At first, the transcripts were carefully read, and data were coded. The data-display and reduction process was conducted at a desk after all data were collected.

## Results

### Socio-demographic characteristics

Of the 550 health workers, 500 returned completed responses with a 90.9% response rate. Fifty-nine percent of the participants were married, 38.2% single, and the rest divorced. Most of the respondents, 308 (61.6%), were females. The mean age of the participants was 29 with SD ±5 years. The majority of the participants, 209 (41.8%), were nurses, followed by 165(33%) health extension workers. The rest 65 (13%) were health officers, and 61 (12.2%) were midwives. About 231 (46.2%) health workers were diploma graduates. The majority of the health workers, 468 (93.6%), had less than 10 years’ of work experience (Table 1).

**Table 1:** Socio-demographic characteristics of health workers at public health facilities of North Gondar zone, northwest Ethiopia, 2017 (n=500)

Characteristics	Number	Percentage
Sex		
Male	192	38.4
Female	308	61.6
Age		
< 29 years	297	59.4
30–39 years	178	35.6
≥ 40 years	25	5
Marital status		
Single	191	38.2
Married	295	59.0
Divorced	14	2.8
Profession		
Health extension	165	33
Midwife	61	12.2
Nurse	209	41.8
Health officer	65	13.
Educational status		
Certificate	114	22.8
Diploma	231	46.2
Degree	155	31
Work experience		
1–10 years	468	93.6
≥ 11 years	32	6.4
Income per month (ETB)		
1000–4000	358	71.6
4001–7000	124	24.8
> 7000	18	3.6

Twenty seven Ethiopian Birr (ETB) = 1 US$

### Growth monitoring practice of health workers

In this study, the prevalence of GM practice was 50.4%. Most, 465 (93%), of the respondents said GM was practiced in their health centers. Three hundred fifty-one (70.2%) of the participants undressed children before weighing to get accurate figures, 454 (90.8%) made use of growth charts, and 206 (41.2%) plotted the weight of children on growth charts in the health centers (Table 2).

**Table 2:** Practice of growth monitoring among health workers at public health facilities of North Gondar zone, northwest Ethiopia, 2017 (*n* = 500)

Characteristics	Frequency	Percentage (%)
Practiced in your health center		
Yes	465	93
No	35	7
Growth chart is used		
Yes	454	90.8
No	46	9.2
Growth card is used		
Yes	299	59.8
No	201	40.2
Undressed the child		
Yes	351	70.2
No	149	29.8
Clean the scale after each child is weighed		
Yes	307	61.4
No	193	38.6
Plotting of children’s ages and weights		
Yes	206	41.2
No	294	58.8
Interpretation of growth curve for each child		
Yes	341	68.2
No	159	31.8
Mothers/caregivers are counseled if need be		
Yes	427	85.4
No	73	14.6
Check the accuracy of weight scale		
Yes	401	80.2
No	99	19.8
Mothers are part of growth monitoring sessions		
Yes	436	87.2
No	64	12.8

### Knowledge and attitude about growth monitoring

Half of the study participants (50.4%) managed to achieve the defined acceptable total knowledge score of 75%. Of the health workers, 100 and 99.8% were aware of GM and the purpose of GM, respectively, while 464 (92.8%) agreed that children between 0 and 2 years should be monitored every month (Table 3).

**Table 3:** Knowledge of growth monitoring among health workers at public health facilities of North Gondar zone, northwest Ethiopia, 2017 (*n* = 500)

Characteristics	Frequency	Percent %
Knowing about the meaning of GM		
Yes	194	38.4
No	306	61.2
Knowing about the purpose of growth chart		
Yes	499	99.8
No	1	0.2
Knowing the equipment of GM		
Yes	218	43.6
No	282	56.4
Knowing correct order of GM		
Yes	434	86.8
No	66	13.2
The nearest weight measurement you recorded		
Yes	212	42.4
No	288	57.6
Indicate deviation of plotted line above the upper reference curve		
Yes	454	90.8
No	46	9.2
Indicate deviation of plotted line below the lower reference curve		
Yes	344	68.8
No	156	31.2
The interpretation of a plotted horizontal line after sickness of the child		
Yes	344	68.8
No	156	31.2
The minimum normal birth weight of a child		
Yes	162	32.4
No	338	67.6
GM intervention needed		
Yes	421	84.2
No	79	15.8
Knowing the GM frequency of children		
Yes	464	92.8
No	36	7.2

*GM* growth monitoring

Almost 58% of the health workers had a favorable attitude towards GM, and 489 (97.8%) held it was necessary for every child, while 486 (97.2%) believed that GM was effective in preventing childhood malnutrition. Of the health workers, 457 (91.4%) had the opinion that GM was necessary not only for the sick but also for the healthy (Table 4).

**Table 4:** Attitude towards growth monitoring practice of health workers at public health facilities of North Gondar zone, northwest Ethiopia 2017 (*n* = 500)

Characteristics	Frequency	Percentage (%)
GM is necessary for every child		
Agree	489	97.8
Disagree	11	2.2
Weighing the child	451	90.2
Agree	451	90.2
Disagree	49	9.8
The process of GM		
Agree	286	57.2
Disagree	214	42.8
Effective to prevent child malnutrition		
Agree	486	97.2
Disagree	14	2.8
Mothers involvement		
Agree	407	81.4
Disagree	93	18.6
GM is burdensome		
Agree	145	29
Disagree	355	71
Used for sick children		
Agree	43	8.6
Disagree	457	91.4
Growth chart and Growth card are useful		
Agree	444	88.8
Disagree	56	11.2
Counseling and interventions		
Agree	423	84.6
Disagree	77	25.4
Training enhance GM		
Agree	483	96.6
Disagree	17	3.4

*GM* growth monitoring

### Training of health workers and supportive supervision

Three hundred fifteen (63%) of the health workers took GM or integrated the management of the newborn and childhood illness training. Out of the trained workers, 235 (74.5%), 202 (64%), 240 (76%), and 230 (72.9%), respectively, said that the training focused on weighing skills, plotting techniques, child feeding counseling methods, and nutrition education.

### Workload and availability of logistic supplies

Four hundred eleven (82.2%) of the respondents saw less than 25 children per day, and the workload reflected ideal practices. Four hundred seventy-five (95%) agreed that the ideal number of patients per day in order to do all GM activities was less than 25. Two hundred sixty-two (52.2%) of the health workers reported that there was lack of GM equipment in their health facilities. Of the 260 health workers, nearly 26 (10%) reported lack of weight scales, 175 (67.3%) lack of family health care cards, 64 (24.7%) lack of stationary materials, and 236(90.7%) reported that there were no pamphlets which promote GM.

### Factors associated with growth monitoring practice

A bivariate analysis was performed to test the associations between GM practice and independent variables, like age, sex, marital status, profession, educational status, work experience, supportive supervision, availability of equipment, training, knowledge, attitude, and workload. The profession, educational status, work experience, availability of GM equipment and attitude were significantly associated with GM with a *p*-value of 0.2.

Variables with less than 0.2 *p*-values were also fitted for the multivariate analysis. In the multivariate logistic regression analysis variables, such as professions, educational status, work experience, attitude, and the availability of logistics were significantly associated with GM practice. GM practice was 0.42 times less likely among midwives compared to HEW (AOR = 0.42, 95%CI: 0.19, 0.94). The odds of GM practice were 2.20 times more likely among diploma graduate health workers compared to certificate holders (AOR = 2.20, 95%CI: 1.09, 4.45). Health workers who had work experience 11 or more years were 4 times more likely practicing GM compared to health workers who had less than 11 of years work experience (AOR = 4.27, 95%CI: 1.7, 10.72). The odds of GM practice was 1.52 (AOR = 1.52, 95%CI: 1.05, 2.20) times more likely among health workers who had adequate logistics and supplies compared to those who had no provisions. GM practice was 0.32 times less likely among health workers who had favorable attitude compared to those who had no such attitude [AOR = 0.68; 95CI:0.47, 0.98] (Table 5).

**Table 5:** Bivariate and multivariate logistic regression of growth monitoring practice at public health facilities of North Gondar zone, northwest Ethiopia,2017 (*n* = 500)

Variables	Practice of GM		Crude OR (95%CI)	Adjusted OR (95%CI)
	Good	Poor		
Profession				
Health extension	86	79	1	1
Midwife	25	36	0.64(0.35,1.16)	0.42 (0.19,0.94)*
Nurse	105	104	0.93(0.62,1.40)	0.62 (0.31,1.22)
Health officer	36	29	1.14(0.64,2.03)	1.07 (0.43,2.61)
Educational status				
Certificate	52	62	1	1
Diploma	130	101	1.53(0.98,2.41)	2.20 (1.09,4.45)*
Degree	70	85	0.99(0.60,1.60)	1.31 (0.55,3.10)
Work experience				
1–10 years	226	242	1	1
≥ 11 years	26	6	4.64(1.88,11.48)	4.27 (1.70,10.72)*
GM equipments				
Not available	118	143	1	1
Available	134	105	1.55(1.09,2.20)	1.52 (1.05,2.20)*
Attitude				
Unfavorable attitude	116	96	1	1
Favorable attitude	136	152	0.74(0.52,1.06)	0.68 (0.47,0.98)*

*Statistically significant at *P* value < 0.05, *COR* Crude odd ratio, *AOR* Adjusted odd ratio, *GM* growth monitoring

### Result of the observation

In the observed health facilities, most of the workers used weight scales made from locally available materials, like basin, and all health workers were trained on how to use such available materials for weight scales, but the scales were not tarred and checked before weighing.

In all the health facilities, weighing scales correctly hung from strong supports when children were placed in the weight scale. Of the observed health workers, only 4 adjusted the scale needle to zero before weighing. Nine health workers waited for the needle to stop wobbling before taking the reading; five health workers suspended the scale at eye level to read easily, and only 3 children were undressed. In the 10 observed health facilities, seven health workers had discussion sessions with mothers/guardians about children’s conditions.

All of the observed workers in all facilities appropriately filled date of entry, name of child, and date of birth; however, dates of appointments were not recorded on the charts; patients were just told when visit workers. Furthermore, did not plot and link the weights of the children with the respective ages. The registration books, prepared by hand did not contain full information about children; thus, it was difficult to find registration numbers when the children came back for GM follow ups because the child was registered as new every time.

### In-depth interview results

Barriers to GM practice in health facilities:

A 29 year old health center manager said, *“….Field supervision was conducted only during the outreach session and when problems were raised by the community.”*

A 32 year old health center manager stated, *“….Growth monitoring practice increases waiting time and decreases client satisfaction at health facilities.”*

A 33 year old health center manager complained, *“….There was a shortage of weight scale for children less than six months and lack of trained health workers for making weighing bags from locally available materials.”*

A 29 year old health facility manager pointed out, “….*There was lack of cooperation of mothers and lost to follow up between consecutive appointments. Mothers did not come back for consecutive weighing unless children got ill or vaccination was announced.”*

A 33 year old health facility manager said, *“….One staff member could not perform multiple tasks at a time. Weighing, recording, and plotting of weight on the card do not allow health workers to give attention to the growth monitoring program, and some of the health workers lack awareness about the program.”*

## Discussion

The prevalence of GM practice among health workers was 50.4%. This finding is in line with that of a study done in Tigray region (53.6%) [14], perhaps due to the similarities of health facility setups, accessibility of GM equipment, and workload. However, this result was lower than that of a study done in Ghana (70.0%) [21]. The variation could be due to socio-cultural differences and the accessibility of GM materials.

Profession, educational status, work experience, availability of materials, and attitude were significantly associated with GM practice among health workers. In-depth interviews showed that lack of training, low motivation and commitment of health workers, and low community participation were problems faced during GM practice.

Growth monitoring practice was more likely among midwives compared to health extension workers. The reason might be that midwives had good knowledge about the importance of GM with more chances to work in logistically better health centers. This was supported by the qualitative finding. Diploma graduate health workers were more likely to practice GM compared to certificate owners. The reason might be knowledge gap and the degree of accessibility of GM materials. When educational status increased, the chance of working at better-equipped health facilities also increased, and the chance of getting training was high. This result was also supported by the qualitative results.

In this study, more experienced health workers were more likely to practice GM than less experienced ones. The reason might be that in Ethiopia, GM skills are mostly developed through experience and training. However, a study done in South Africa showed that experience inversely affected the usage of GM in comparison with less experienced health workers [13]. The reason might be that senior health workers see more patients per day, and this causes workers not to use growth charts regularly.

Health workers who had favorable attitude were less likely to practice GM than their counterparts. This finding is in line with those of studies done in Ethiopia [14], South Africa [13], and Nigeria [17]. The reason might be that workload has been frequently associated with high levels of stress, exhaustion, and job dissatisfaction, resulting in lower job performance [22]. In this study, most of the health personnel saw more than 25 patients per day which might have an effect on low practice of GM even if they had the attitude. In addition, out of the health workers with good attitude, about 62.6% were highly loaded by different activities and 52.2%, had no access to equipment in their health facilities to practice GM. This was supported by the result of the qualitative findings.

Health workers who had better logistic supplies at their health facilities were more likely to practice GM. This result was supported by those of studies done in Ethiopia [15] and Zambia [18]. The possible explanation might be that health facilities that had adequate GM equipment encouraged health professionals to practice better services.

The observational result showed that most of the health workers did not read weight scale at eye level, remove soaked diapers, and calibrate the scale every week by a known mass. Furthermore, they did not connect dots on the chart, plot the weight of the child every month on the card and had no mechanisms to trace children lost to follow up. Our qualitative finding was supported by those of similar studies done in Brazil [23] and Zambia [24]. The possible reason might be lack of training, motivation, and overload.

Even though mothers were given nutritional counseling after weighing their children, health workers did not counsel them based on the age of the children and the growth curve position. This finding was supported by those of studies done in Ethiopia [20] and Zambia [24]. The reason might be workload, lack of training about counseling techniques, and mothers’ impatience to wait and get counseling.

The in-depth interview showed that lack of cooperation and lost to follow up between consecutive appointments of caregivers, high workload, low commitment or motivation among health workers were the problems against GM. This study was supported by studies conducted in Ethiopia [15] and Nigeria [17]. The probable reason might be low motivation of health workers and lack of involvement of communities.

### Strength and limitation of the study

The strength of this study is its being mixed; however, it has its own limitation in that it didn’t include mothers and health workers and couldn’t use audio-recording equipment for the in-depth interview. The authors recommend further study to investigate the association between attitude and GM practices among health workers.

## Conclusion

In this study, the magnitude of GM practice among health workers was low. Profession, educational status, work experience, attitude, and availability of materials were significantly associated with the practice. Therefore, giving training about growth monitoring, fulfilling logistic requirements and equipment are important to improve growth monitoring.

## Abbreviations

AOR: Adjusted Odds Ratio
COR: Crude Odd Ratio
EDHS: Ethiopian Demographic and Health Survey
GM: Growth Monitoring;
HEW: Health Extension Workers
KAP: Knowledge, Attitude and Practice
PH: Primary Health Worker
UNICEF: United Nation International Children’s Fund
WHO: World Health Organization

## References

[1] Hall DM (2000). Growth monitoring. Arch Dis Child.

[2] Ashworth A, Shrimpton R, Jamil K (2008). Growth monitoring and promotion: review of evidence of impact. Matern Child Nutr.

[3] Dixon RA (1991). Monitoring the growth of the world's children. Ann Trop Paediatr.

[4] Tremlett G, Lovel H, Morley D. Guidelines for the design of national weight-for-age growth charts. Assign Child. 1983;61/62:143–75.

[5] Mangasaryan N, Arabi M, Schultink W (2011). Revisiting the concept of growth monitoring and its possible role in community-based nutrition programs. Food Nutr Bull.

[6] Unicef/WHO. Levels and trends in child malnutrition. eSocialSciences; 2018. https://www.who.int/nutgrowthdb/2018-jme-brochure.pdf.

[7] Save the Children. Ethiopia National Nutrition Strategy Review and Analysis of Progress and Gaps. UK; 2009. http://outcomestories.ifpri.info/2015/06/15/ethiopia-nationalnutrition-strategy/

[8] ICF, C.S.A.C.E.a., Ethiopia Demographic and Health Survey (2016). Addis Ababa, Ethiopia, and Rockville, Maryland.

[9] Federal Democratic Republic of Ethiopia MoH. Government of Ethiopia National Nutrition Program, 2016-2020. Addis Ababa, Ethiopia; 2016. https://eeas.europa.eu/sites/eeas/files/nnp2_pdf.pdf.

[10] Pelletier DL (2011). The nutrition policy process: the role of strategic capacity in advancing national nutrition agendas. Food Nutr Bull.

[11] UNICEF and UNICEF (2008). Experts’ consultation on growth monitoring and promotion strategies: Program guidance for a way forward. Recommendations from a technical consultation.

[12] Wright CM (2012). Designing new UK-WHO growth charts: implications for health staff use and understanding of charts and growth monitoring. Matern Child Nutr.

[13] Smith S, Reji E (2015). Doctors’ attitudes to and knowledge and usage of growth charts. S Afr Fam Pract.

[14] Baraki TM, Gebru AA, and Belay DG. Knowledge attitude and practice of health extension workers towards growth monitoring and promotion program in Tigray region, Ethiopia. Eur J Biomed Pharm Sci. 2018;3(4):55–64.

[15] Bilal SM (2014). Practices and challenges of growth monitoring and promotion in Ethiopia: a qualitative study. J Health Popul Nutr.

[16] Kapil U, Joshi A, Nayar D (1994). Utility of growth monitoring: its relevance in the promotion of child health. Indian Pediatr.

[17] Iyanuoluwa O-BA, Esther A-OO, Adeleye AA (2011). Primary health care workers’ role in monitoring children's growth and development in Nigeria, West Africa. Global J Health Sci.

[18] Charlton KE, Kawana BM, Hendricks MK (2009). An assessment of the effectiveness of growth monitoring and promotion practices in the Lusaka district of Zambia. Nutrition.

[19] Bosu WK (2012). A comprehensive review of the policy and programmatic response to chronic non-communicable disease in Ghana. Ghana Med J.

[20] Tuobom Debuo Debora (2017). Caregivers Knowledge, Attitude and Practices on Child Growth Monitoring and Promotion Activities in Lawra District, Upper West Region of Ghana. Science Journal of Public Health.

[21] Debuo TD (2017). Caregivers knowledge, attitude and practices on child growth monitoring and promotion activities in Lawra District, upper west region of Ghana< previous article next article science. J Public Health.

[22] Hauck EL, Snyder LA, Cox-Fuenzalida L-E (2008). Workload variability and social support: effects on stress and performance. Curr Psychol.

[23] de Almeida AC (2016). Uso de instrumento de acompanhamento do crescimento e desenvolvimento da criança no Brasil–Revisão sistemática de literatura. Revista Paulista de Pediatria.

[24] Msefula D (1993). How can growth monitoring and special care of underweight children be improved in Zambia?. Trop Dr.

